# Pandemic (H1N1) 2009 Virus Circulating in Pigs, Guangxi, China

**DOI:** 10.3201/eid1802.111346

**Published:** 2012-02

**Authors:** Jian-Hua Yan, Yi Xiong, Chun-Hua Yi, Xiang-Xiang Sun, Qi-Song He, Wei Fu, Xian-Kun Xu, Jia-Xia Jiang, Lin Ma, Qi Liu

**Affiliations:** Guangxi University, Guangxi, People’s Republic of China (J.-H. Yan, C.-H Yi, X.-X. Sun, Q.-S He, J.-X Jiang, Q. Liu);; Guangxi Center for Animal Disease Control and Prevention, Guangxi (Y. Xiong, W. Fu, X.-K. Xu, L. Ma)

**Keywords:** swine influenza, genetic analysis, pandemic (H1N1) 2009 influenza, influenza, viruses, China

**To the Editor:** A novel swine-originated influenza A virus known as pandemic (H1N1) 2009 was first isolated from humans in Mexico in April 2009 (*1*), and a worldwide pandemic followed, which affected >214 countries and resulted in >18,000 deaths (*2*). In August 2010, the World Health Organization stated that the pandemic caused by this virus had ended. As this virus emerged, animals, including swine, turkeys, ferrets, cats, and cheetahs, were found to have been infected (*3*). In addition, transmission from humans to pigs in porcine herds has been reported (*4*).

Swine influenza A virus (SIV) belongs to the family *Orthomyxoviridae* and is a causative agent of respiratory disease in pigs (*5*). Currently, 3 subtypes of influenza viruses are circulating in the swine population globally: H1N1, H3N2, and H1N2 (*6**,**7*). Although pigs can be simultaneously infected with avian influenza viruses and human influenza viruses, the viruses can exchange genes and produce new variants, which suggests that pigs have become mixing vessels for influenza viruses (*8*). Pandemic (H1N1) 2009, caused by a virus usually circulating in pigs in Europe and Asia, is a triple hybrid that contains swine, human, and avian virus gene segments, which further emphasizes that SIVs pose a serious threat to public health. We describe an outbreak of pandemic (H1N1) 2009 virus, which was isolated from a pig farm in Guangxi Province, People’s Republic of China, and report the consequences of subsequent epidemiologic studies.

In January 2011, an outbreak of severe respiratory problems occurred in pigs on a pig farm. Nine hundred growing and fattening pigs exhibited clinical signs of influenza, including fever, cough, runny nose, loss of appetite, lethargy, edema, watery eyes, conjunctivitis, diarrhea, and vomiting. The incidence rate was ≈80%, and the death rate was 22%. The outbreak lasted ≈2 weeks. However, no outbreak of respiratory disease occurred in other pig farms in the same area simultaneously, and no evidence of human-to-pig transmission was found.

We collected lung samples from 3 dead pigs with underlying illness for reverse transcription PCR and virus isolation in 10-day-old specific pathogen–free embryonated chicken eggs. Viral RNA was extracted from the tissue suspension and allantoic fluids. Virus isolation was assessed by hemagglutination inhibition (HI) assay and neuraminidase inhibition assay by using a panel of reference serum samples (National Reference Laboratory for Avian Influenza, Harbin Veterinary Research Institute, Harbin City, China). Later, 3 viruses were isolated. All of them had hemagglutination (HA) activity, and the HA titers ranged from 128–256. The HA-positive isolates were further identified as subtype H1N1. Subsequently, nucleotide sequences of the 8 viral genes were amplified and sequenced (GenBank accession nos. JN222372–JN222379). This analysis showed high identities to a pandemic strains A/California/04/2009 (H1N1), hemagglutinin (99.2%), neuraminidase (99.1%), matrix (99.3%), nucleoprotein (99.5%), nonstructural protein (98.5%), polymerase acidic protein (98.5%), polymerase basic protein 1 (99.7%), and polymerase basic protein 2 (99.6%) genes. Bacteria were cultured from spleen, liver, and heart-blood samples from 5 pigs. Four pigs were infected with porcine streptococci, and 1 pig with mild symptoms was negative for the bacterium.

We sought to gain more insight into the epidemiology of pandemic (H1N1) 2009 virus in Guangxi Province and collected 600 bronchial swab samples and 200 blood serum samples when pigs were slaughtered every month from February through June 2011. The samples were used to isolate virus in 10-day-old specific pathogen–free embryonated chicken eggs, and then the virus isolates were subjected to sequencing of a partial genome of the HA gene. Overall, we obtained 10 strains of subtype H1N1 influenza virus, including 5 strains of classic swine H1N1, 3 strains of Eurasian avianlike H1N1, and 2 strains of pandemic (H1N1) 2009 virus, which were derived from 3,000 bronchial swab samples.

In addition, a serologic survey was implemented by using HI testing with pandemic (H1N1) 2009 virus and SIV (H1N1) antigens. Serologic studies showed that 251 of the 1,000 samples tested had positive HI titers for pandemic (H1N1) 2009 virus, and 248 of these samples had positive HI titers for SIV (H1N1). Notably, cross-reactivity of pandemic (H1N1) 2009 virus between H1 subtype viruses has been reported recently in pigs (*9*). However, the higher rate of positive test results indicated that swine serum samples contained antibodies against pandemic (H1N1) 2009 virus.

Our findings strengthened previous data by showing that growing and fattening pigs are susceptible to infection of pandemic (H1N1) 2009 virus (*4*). Analysis of the complete genome sequence of the subtype H1N1 isolates suggests that no gene reassortment occurred. The results of serologic studies demonstrated that uninfected pig farms are also susceptible to pandemic (H1N1) 2009 virus infection. Our results suggest that the pandemic virus is currently circulating in swine populations and posing a challenge to pigs in southern China. Increasing serologic surveillance of pigs for prevention and better control of pandemic influenza is urgently needed in China.
